# Design and Application of Intelligent Local Anesthetic Nanoformulations

**DOI:** 10.3390/pharmaceutics18030289

**Published:** 2026-02-27

**Authors:** Peng Ke, Yuying Li, Min Han, Xiaodan Wu

**Affiliations:** 1 Department of Anesthesiology, Fujian Provincial Hospital, Fuzhou University Affiliated Provincial Hospital, Shengli Clinical Medical College of Fujian Medical University, Fuzhou 350001, China; kepeng@fzu.edu.cn (P.K.); sy1668@fzu.edu.cn (Y.L.); 2 Institute of Pharmaceutics, College of Pharmaceutical Sciences, Zhejiang University, Hangzhou 310058, China

**Keywords:** local anesthetics, intelligent drug delivery, on-demand analgesia, triggered release, nanotechnology

## Abstract

Local analgesia is a prevalent and cost-effective pain management strategy with minimal systemic side effects. At the same time, nanotechnology has been employed to achieve the sustained release of drugs to prolong the duration of local anesthetics (LAs). However, these traditional nanoformulations lack responsiveness and thus cannot achieve precise pain relief through on-demand administration. The emergence of “intelligent” nanosystems with stimulus-response capabilities has opened up new prospects in pain control. This review summarizes recent advancements in the design and application of triggerable LAs nanoformulations that can be activated by external stimuli such as light, ultrasound, or heat. These systems facilitate precise, patient-specific pain management, transforming the clinical approach from long-term suppression to controllable and on-demand relief. Finally, we discussed the current challenges and future prospects. A deeper understanding of this field will facilitate the development of superior analgesic solutions and ultimately improve the treatment outcomes for patients.

## 1. Introduction

LAs, as reversible inhibitors of voltage-gated sodium channels [1], play an indispensable role in modern pain management. By blocking the transmission of nerve signals, they can specifically inhibit the harmful signals with high safety. Therefore, local anesthetic analgesia has become an indispensable and crucial method for enhancing recovery after surgery (ERAS) [2]. These LAs can be classified into short-acting (such as procaine), medium-acting (such as lidocaine, mepivacaine), and long-acting (such as bupivacaine, ropivacaine) compounds based on their duration of action [3]. Although LAs provide effective pain relief, a major limitation still exists: the duration of analgesia is insufficient. While adjunctive medications such as opioids [4] or dexmedetomidine [5] can moderately prolong analgesic effects, and continuous catheter techniques offer an alternative approach, these methods introduce complex issues including side effects, high costs, and risks of infection or tissue damage [6,7,8]. Consequently, achieving sustained and high-quality analgesia with a single dose remains a challenging goal.

Nanoscale drug carriers (NDCs) offer a transformative solution by encapsulating therapeutic drugs to modulate their release kinetics, enhance bioavailability, improve targeting, and reduce systemic toxicity [9]. The multifunctionality of NDCs is evidenced by their success across numerous fields, ranging from attenuating oxidative stress and inflammation [10] to enhancing anti-tumor effects [11] and stabilizing the release of hypoglycemic drugs in diabetic patients [12]. In the field of LAs, the primary clinical demand is to extend their duration of action. In this regard, nanotechnology has achieved significant outcomes. Formulations such as liposomes [13,14], microspheres [15], and polymeric nanoparticles [16] have been engineered to deliver prolonged analgesic effects, with the clinically applied liposomal bupivacaine (Exparel^®^) serving as a prime example [17]. Beyond their sustained-release properties, NDCs also enhance neural permeability and promote local drug retention, thereby further optimizing analgesic efficacy and surgical feasibility [18,19]. However, as clinical expectations continue to evolve, it has become increasingly recognized that merely prolonging analgesia duration is insufficient. The dynamic nature of pain typically manifests as intermittent episodes rather than continuous presence, necessitating an on-demand analgesic approach. Unnecessary continuous nerve blocks offer no clinical benefit and may carry risks. This gap highlights the urgent need for a new generation of on-demand analgesic systems capable of activation in response to specific pain stimuli.

Triggerable nanoformulations represent a frontier solution for on-demand pain relief. These intelligently designed systems, often known as remotely triggerable drug delivery systems (RTDDS), utilize smart polymeric nanomaterials that release drugs in response to external or internal stimuli like temperature, pH, light, or ultrasound [20]. RTDDS has achieved significant progress in treating various diseases. For instance, glucose-responsive nanoparticles have been engineered to release insulin directly when blood glucose levels rise [21]. In oncology, enzyme-triggered nanoparticles activate within the acidic tumor microenvironment to release anti-tumor drugs [22]. This principle of stimulus-responsive release is directly applicable to LAs. By delivering LAs only when pain stimuli occur, RTDDS have transformed postoperative pain management, providing personalized relief while minimizing drug exposure.

The ongoing development of RTDDS is providing a growing array of material alternatives, significantly accelerating the development of intelligent LAs [23]. Given the substantial research achievements in the field of LAs, numerous review articles have systematically summarized advances in nanoparticle formulations for pain management. These prior publications primarily focused on the administration routes, animal models, and evaluation methods of local anesthetic delivery systems [24]. Additional studies have explored carrier systems with varying sizes and structural morphologies, such as nanocapsules and nanospheres [25]. Local anesthetic delivery systems based on natural polymers have also garnered significant attention [26]. Relevant reviews encompass experimental validation, animal studies, and clinical trials, with particular emphasis on hydrogel and bupivacaine formulations [27,28]. Despite these significant contributions, there is a lack of systematic discussions from the perspective of triggering mechanisms. Existing reviews have not systematically compared the roles of different triggering modes in LAs formulations, nor have they thoroughly evaluated their mechanisms of action, material safety, or systematically analyzed core barriers hindering clinical translation.

**Figure 1:**
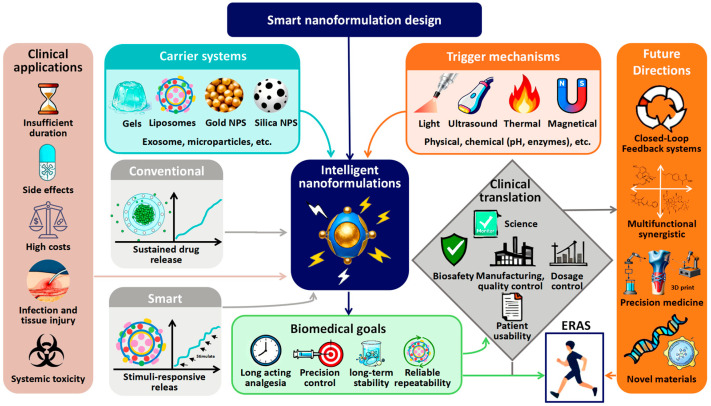
Schematic diagram of the scope and structure of this review. This figure illustrates the logical framework of the review. Starting from existing clinical local anesthetics, it progresses to traditional nanomedicines and ultimately forms smart nanoformulations responsive to external stimuli. The focus highlights carrier platforms and triggering mechanisms, as well as biological goals and challenges in clinical translation. The ultimate goal is to establish rational design principles to facilitate the clinical translation of on-demand analgesia. The authors created this figure using WPS Presentation (12.1.0.24657).

Given the well-established clinical role of LAs, the successful development of their intelligent forms holds considerable potential to redefine perioperative care and pain management. This review is structured to provide a critical appraisal of recent advances in triggerable nanoformulations for local anesthesia. We will systematically explore systems responsive to the most prominent physical stimuli, including light, ultrasound, magnetic fields, and temperature. A primary focus will be placed on the dominant lipid-based and polymeric platforms, such as liposomes and nanoparticles. For each category, we will meticulously compare the synthesis techniques, trigger-controlled release profiles, resultant analgesic efficacy, and biocompatibility. Our objective is to consolidate the current understanding and establish rational design principles to guide the development of future triggerable LAs, thereby accelerating the clinical translation of intelligent analgesia. For clarity, Figure 1 provides an overview of the scope and logical framework of this review. Beginning with the established clinical roles of local anesthetics, the diagram illustrates the evolutionary path toward smart nanomedicines, key trigger mechanisms and delivery platforms currently under investigation, and the ultimate goal of establishing rational design principles for clinical translation.

## 2. Current Clinical Applications of LAs

LAs form a cornerstone of modern analgesic therapy, primarily due to their rapid onset, targeted pain-blocking capabilities, and minimal impact on patient consciousness or motor function [29]. A critical advantage of LAs lies in their excellent safety profile, characterized by negligible effects on vital physiological functions such as respiration, heart rate, and blood pressure [30,31]. The clinical utility of LAs is further demonstrated by their remarkable flexibility in administration routes. The following section will detail the primary anesthetic techniques according to these common sites of administration and application methods.

### 2.1. Topical Anesthesia

Topical anesthesia involves the direct application of LAs to mucosal or skin surfaces, achieving surface anesthesia by blocking underlying nerve endings [32]. This technique is particularly suitable for superficial procedures involving the eyes, nose, mouth, and genitourinary tract. Pharyngeal spraying before otolaryngological surgery represents a common example of topical anesthesia. Topical anesthesia also serves as a critical component of conscious sedation intubation, suppressing airway reflexes and enhancing patient tolerance [33,34]. The primary challenge facing topical anesthesia is the difficulty in effectively retaining anesthetic agents within the target area. On one hand, the fluid nature of the solution causes it to readily migrate away from the application site, necessitating frequent reapplication; on the other hand, the limited tissue penetration capacity of the drugs makes it difficult to achieve the desired depth of anesthesia, thereby compromising the efficacy of the anesthetic effect. To address diverse clinical scenarios, LAs are formulated into various topical preparations, such as lidocaine patches [35] and creams [36], which are extensively utilized for managing postoperative wound pain.

### 2.2. Infiltration Anesthesia

Infiltration anesthesia involves the direct injection of a local anesthetic solution into subcutaneous or specific target tissues to block nerve endings [37]. While this technique provides excellent analgesia, a significant limitation is the rapid diffusion of the drug from the injection site, often requiring higher doses and increasing the risk of systemic toxicity due to vascular absorption [38]. To mitigate these risks and prolong analgesia duration, adjuvants such as epinephrine, dexmedetomidine, or dexamethasone are frequently combined with LAs like lidocaine, ropivacaine, or bupivacaine [39,40,41]. This approach improves patient satisfaction and reduces postoperative complications in brief procedures such as inguinal hernia repair [42]. Consequently, infiltration anesthesia is widely adopted in dental surgery [43] and various diagnostic or therapeutic puncture procedures [44].

### 2.3. Nerve Block

Nerve block entails the injection of LAs near peripheral nerves to achieve conduction anesthesia in specific anatomical regions [45]. This technique has become a primary method for perioperative regional analgesia, offering superior pain control. Combined with opioids as part of a multimodal treatment strategy, it effectively reduces overall opioid consumption and associated addiction risks [46]. Among the amide LAs, ropivacaine is particularly favored for regional analgesia due to its clinically valuable characteristic of differential sensory and motor block [47]. It is worth noting that nerve block techniques also carry the risk of procedural failure, nerve injury, or toxic reactions to LAs. Ultrasound-guided nerve blocks enable real-time visualization of precise needle placement and local anesthetic deposition, significantly enhancing procedural efficacy and safety [48]. This advancement is supported by various successful clinical cases, such as interscalene brachial plexus block after shoulder arthroscopy [49], transversus abdominis plane (TAP) block after cesarean section [50], and erector spinae plane (ESP) block for pain management after thoracic surgery [51]. Numerous other types of blocks, including those targeting the femoral nerve, fascia iliaca, and cervical plexus, are also widely employed in clinical practice, underscoring the diversity and significance of this analgesic approach.

### 2.4. Intraspinal Anesthesia

Intraspinal anesthesia encompasses several techniques, including subarachnoid anesthesia (spinal anesthesia), epidural anesthesia, combined spinal-epidural anesthesia, and caudal blocks. These procedures involve injecting LAs into the subarachnoid or epidural space, where they act on spinal nerve roots to produce a reversible sensory and motor block in the corresponding innervated regions [52]. This approach is particularly suitable for surgeries involving the lower abdomen and lower extremities, with its most prominent application in cesarean section and labor analgesia [53]. The depth and intensity level of anesthesia are influenced by several factors, such as patient positioning, solution density, injection dosage, and administration speed. While experienced anesthesiologists typically achieve satisfactory analgesia, the technique is not without significant challenges. Critical considerations include the risk of hypotension, respiratory depression, and potential complications from spinal anesthesia toxicity or systemic local anesthetic toxicity.

**Figure 2:**
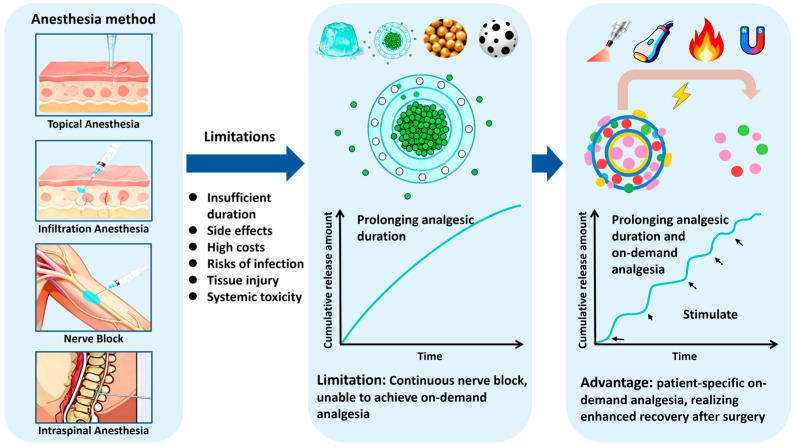
The evolution of LAs to intelligent nanoformulations. Anesthesia method: topical anesthesia: diagram showing drug application on the mucosal surface. Infiltration anesthesia: syringe injecting an anesthetic into subcutaneous tissue with local drug diffusion. Nerve block: Percutaneous injection of a local anesthetic around the nerve. Intraspinal anesthesia: spinal anatomy showing needle insertion into the epidural/subarachnoid space. Challenges: Conventional local anesthetic injections (not nanoformulations) have limited duration, side effects, high costs, risks of infection, tissue injury, systemic toxicity, etc. Traditional nanoformulations (liposomes, microspheres, etc.) exhibit sustained-release characteristics, while their “One-time” release fails to address individual variability and dynamic pain patterns. Next-generation intelligent systems are stimulus-responsive nanocarriers activated by various methods, including NIR light, ultrasound waves, and magnetic fields. External stimuli trigger pulsatile drug release, enabling on-demand and intelligent pain relief. The authors created this figure using WPS Presentation (12.1.0.24657).

Collectively, these established anesthetic modalities are constrained by the inherent limitations of conventional LAs injections (not nanoformulations). While early nanocarriers like liposomes and microspheres have made significant progress in prolonging drug release, they are often designed for a “one-time” and sustained release profile. These formulations, lacking trigger-release capabilities, are termed traditional LAs nanoformulations. This passive approach fails to account for individual patient variability and the dynamic nature of postoperative pain, leaving it ineffective for on-demand analgesia. These shortcomings, coupled with issues of rapid metabolism and catheter-related burdens, create a considerable unmet clinical need. Consequently, there is a compelling rationale for developing intelligent formulations that move beyond simple sustained release to provide responsive, on-demand pain control. Figure 2 illustrates the evolution of local anesthesia from conventional LAs injections to intelligent nanoformulations, which helps to understand the urgency of on-demand analgesia.

## 3. Remotely Triggerable Drug Delivery Systems

RTDDS represent an advanced class of nanoformulations designed to release therapeutic agents in response to specific external or internal stimuli [54]. This system directly addresses the inherent limitations of conventional drug delivery methods by enabling the real-time, on-demand modulation of drug bioavailability and pharmacological effects [55]. The advancement of RTDDS stems from their potential to achieve intelligent, precise, and efficient drug delivery. An ideal RTDDS platform possesses key characteristics, including being non-invasive or minimally invasive, featuring a trigger-release mechanism with high sensitivity and excellent repeatability and enabling precise control over the dose and duration of each release event. Furthermore, targeting specificity, sufficient drug-loading capacity, and minimal premature leakage under non-triggering conditions are all essential for the successful clinical translation of this approach.

The advancement of nanotechnology has catalyzed the exploration of RTDDS across various clinical fields. Based on the nature of the triggering stimulus, RTDDS are classified into active (closed-loop) triggering systems and passive (open-loop) triggering systems. Active triggering systems operate autonomously as closed-loop systems, initiating drug release in response to endogenous physiological changes (such as fluctuations in pH, temperature, glucose levels, or enzyme activity) without requiring external intervention. A representative example is glucose-responsive membranes, which enhance insulin permeation under hyperglycemia conditions [56]. Similarly, nano-peptides can be designed to self-assemble upon encountering elevated levels of specific enzymes (such as MMP9) in the tumor microenvironment, achieving targeted anti-tumor effects [57]. This autonomous functionality represents high intelligence, yet its application is inherently confined to pathological conditions possessing unique and exploitable physiological signals, thereby limiting its versatility across different therapeutic areas. We have provided a comparative analysis of the current progress and challenges associated with local anesthetic injections, conventional nanoformulations, and intelligent nanoformulations in Table 1.

In contrast, passive triggering systems function as open-loop control systems. Drug release depends on an external device that emits specific stimulus signals, such as near-infrared (NIR) light, ultrasound, or a magnetic field, thereby inducing the impulsive release of the drug [58]. Since this release mechanism relies on externally applied signals with high spatiotemporal precision, it remains independent of the internal physiological environment. This characteristic has led to passive triggering systems, particularly those sensitive to physical stimuli, becoming the primary focus of this review within the field of LAs.

## 4. The Design Cornerstones of Intelligent Anesthesia Systems: Materials and Triggering Mechanisms

The development of intelligent LAs RTDDS relies on the synergistic integration of responsive materials with precise triggering mechanisms. RTTDS enable the on-demand activation and release of LAs in response to specific external stimuli. To achieve this goal, a wide variety of nanoformulations have been explored, each with unique and complex preparation methods. Therefore, designing ideal intelligent nanoparticles requires a critical evaluation of the advantages and limitations inherent to each material platform and synthesis method. Indeed, researchers have multiple material options for constructing RTDDS [59].

### 4.1. Core Delivery Systems: Why Are Gels an Ideal Platform?

Among various nanocarriers, gel-based systems (particularly hydrogels and organogels) serve as ideal platforms for local anesthesia [60]. The advantages of gel-based systems include:

- High drug loading capacity and sustained baseline release: The porous three-dimensional network can encapsulate large quantities of hydrophilic or hydrophobic LAs, enabling sustained baseline drug release. This release effect can be enhanced through triggered bursts [61].
- Versatile and tunable responsiveness: The chemical and physical structure of gels can be engineered to respond to various external stimuli (e.g., heat, light, ultrasound). This responsiveness is achieved through reversible sol–gel transitions or volume changes, enabling the release of encapsulated substances [62].
- Excellent biocompatibility and injectable sustained-release formulations: Many hydrogel precursor materials exhibit biocompatibility and can be designed as injectable liquid formulations. Upon administration, they gelify to form stable, localized sustained-release systems at the target site (e.g., perineurally), minimizing systemic diffusion while prolonging residence time at the intended location [63].
- Protection of encapsulated drugs: The gel matrix shields LAs from premature enzymatic degradation or rapid clearance, enhancing their stability and bioavailability [64].

Traditional nanocarriers, such as polymeric nanoparticles and liposomes, possess well-defined nanostructures, enhanced cellular uptake, and proven clinical translation. Integration of these traditional nanocarriers into gel matrices can further enhance their functionality. Gel-based systems are not substitutes but rather serve as a versatile, foundational platform that synergistically combines high drug loading, superior spatial control, and tunable responsiveness with the specific strengths of nanocarriers. This strategic combination forms an advanced hybrid system, making gel-based composites an ideal and versatile foundation for constructing the next generation of intelligent, on-demand LAs RTTDS.

When constructing hydrogel systems, it is essential to comprehensively consider the mechanical microenvironment at the delivery site, the hydrogel’s resistance to deformation, and how mechanical forces influence the release profile. The administration site of LAs (joint cavity, subcutaneous tissue, perineural space, or between muscle and fascia) is situated within a complex dynamic mechanical environment. This primarily includes micro-movements, compressive loads, tissue sliding friction, and pulsatile compression around nerves. Mechanical factors influence hydrogel integrity and drug release behavior. Micro-movements and friction manifest around joints or nerves, where relative tissue sliding imposes shear stress on hydrogels. Insufficient interfacial adhesion may cause gel-tissue separation or even fragmentation. Research indicates that hydrogels undergo gel-sol phase transitions at shear stresses of approximately 9.04 Pa in most tissues. Therefore, the constructed nanomedicine formulations should meet this mechanical parameter [65]. Additionally, in weight-bearing areas or regions subjected to muscular compression, hydrogels require sufficient compressive modulus to maintain their three-dimensional network structure. Repeated compressive loading may induce network fatigue and microcrack formation, thereby accelerating drug diffusion [66]. Finally, the perineural environment presents unique challenges. Neural tissues are highly sensitive to compression and exhibit significant gliding range. If hydrogels form envelopes around nerves, their stiffness must match that of neural tissues to prevent compression injuries.

Hydrogels prepared as RTDDS should possess suitable rheological properties and deformation resistance. For instance, the hydrogel remains in a dissolved state at 10 °C but rapidly gels within 30 s at the average human body temperature of 37 °C [67]. LAs require administration via syringe, and can be delivered through in situ gelation, wherein the hydrogel forms in response to physiological stimuli after injection. Another approach is shear thinning, where hydrogels exhibit non-Newtonian fluid behavior. During injection, they demonstrate reduced viscosity under high shear rates yet rapidly regain mechanical strength once shear stress is removed. For such shear-thinning systems, viscosity must remain below 1 Pa to ensure smooth injection [68].

Mechanically regulated drug release mechanisms, including pore compression, network deformation, shear-induced release, and force-induced bond cleavage, should be incorporated into nanodrug delivery design. These principles can be leveraged to engineer mechanoresponsive drug delivery systems, such as “on-demand” hydrogels that accelerate drug release with increased movement intensity. The mechanical microenvironment also poses risks of uncontrolled release. When hydrogels exhibit insufficient fatigue resistance, repeated stress loading may cause macroscopic fragmentation, dramatically increasing exposed surface area. Insufficient adhesion at the gel-tissue interface can lead to debonding under micro-movements, forming leakage pathways for drugs. Degradation-induced mechanical property decline may cause hydrogels to prematurely reach yield points, resulting in structural instability [69].

### 4.2. Classification of Triggering Mechanisms and Their Physicochemical Principles

The efficacy of intelligent LAs RTDDS fundamentally depends on their triggering mechanisms. These mechanisms are categorized into two types based on the source of stimulation: external triggering mechanisms, which involve energy fields applied from outside the body, and internal triggering mechanisms, which exploit physiological changes within the disease microenvironment [70]. The following sections will delve into the most prominent external triggering. 

#### 4.2.1. Light-Triggered Drug Release

NIR light typically spans wavelengths in the range of 650–900 nm. It exhibits excellent tissue penetration and low phototoxicity compared to ultraviolet light, making it an ideal external trigger [71]. Light-triggered release primarily operates through a photothermal mechanism, where photons are absorbed by conversion material and converted into localized heat, inducing phase transitions or structural changes in the carrier to release the payload.

Liposomal platforms for photothermal anesthesia: Seminal work by Daniel S. Kohane’s research group has established liposomes as the dominant platform for light-triggered local anesthesia. A foundational system, tetrodotoxin-photosensitizer liposomes (Lipo-PS-TTX), was engineered using the palladium phthalocyanine-based photosensitizer PdPC(OBu)_8_. Following NIR exposure (730 nm, 15 min), this system delivered two distinct, on-demand analgesic effects in addition to a substantial baseline nerve block, demonstrating the feasibility of pulsed drug release [72]. This is particularly significant as it addresses the dynamic nature of postoperative pain, which typically peaks within the initial 48 h [73].

To enhance photothermal efficiency and prolong analgesic effects, researchers incorporated gold nanorods (GNRs) into thermosensitive liposomes. A cold-sensitive liposome loaded with tetrodotoxin and gold nanorods (LTSL-GNR-TTX) system, a remarkable total analgesic duration of 37.4 h, enabling three on-demand releases that further extended analgesia by 14 h [74]. The strategy was further advanced by encapsulating the adjuvant dexmedetomidine alongside tetrodotoxin with GNR-liposomes. This synergistic effect extended trigger-induced analgesia to 13.0 h across four cycles [75,76]. Subsequent innovations focused on improving stimulus sensitivity. A hybrid system combining photosensitizers with GNRs reduced the required irradiation time to just 3 min while maintaining effective on-demand release [77,78]. This evolution highlights a clear pathway toward maximizing therapeutic efficacy while minimizing energy input and patient exposure to external stimuli.

Surface engineering and biosafety of gold nanorods remain critical challenges. The surfactant cetyltrimethylammonium bromide (CTAB), essential for the classical seed-mediated growth method, constitutes a core obstacle to their clinical translation [79]. While CTAB’s bilayer structure confers colloidal stability to GNRs, its cytotoxicity (membrane lysis, ROS induction) and hemolytic effects preclude direct in vivo application. Consequently, any biomedical GNRs must undergo thorough surface modification to replace or shield CTAB. Surface modification strategies include ligand exchange (e.g., mPEG-SH substitution), polymer coating (e.g., polyethylene glycolization), inorganic shell coating (e.g., silica), and lipid bilayer modification. Polyethylene glycolation is currently the most widely adopted method, reducing protein adsorption, extending circulation half-life, and partially shielding CTAB toxicity by forming a steric hindrance layer [80]. However, PEGylation struggles to achieve complete CTAB displacement; residual CTAB may become encapsulated beneath the PEG layer or at the GNR tips, posing a non-negligible risk of long-term slow leakage. In contrast, silica coating physically isolates and completely encapsulates CTAB. CTAB can be extracted during coating, yielding toxicity data significantly superior to PEGylated GNRs. However, thickening the silica shell (>10 nm) may weaken localized surface plasmon resonance effects, and the mechanical mismatch between the rigid shell and neural tissue warrants caution [81].

Significant gaps remain in the current safety data required to advance GNRs toward clinical applications in the perineural setting. The biodistribution of GNRs following perineural administration is an often-overlooked issue, as nanoparticles may be transported retrograde along the intraneuronal lymphatic system or axoplasm to the spinal cord, posing a risk of central nervous system exposure. Therefore, claims of “biocompatibility” based solely on cell viability data are wholly inadequate. Safety evaluation must extend beyond conventional cell viability assays. An ideal assessment system should encompass: (1) electrophysiological function (nerve conduction velocity, compound action potentials); (2) myelin integrity (MBP immunostaining, transmission electron microscopy); (3) long-term tissue response (chronic inflammation, fibrosis); (4) Axonal transport and central exposure risk [82]. Unfortunately, most studies were confined to short-term cell experiments, with minimal coverage of these critical indicators [83]. This review argues that bionic membrane encapsulation strategies combining CTAB complete shielding, tissue mechanical compatibility, and long-term stability represent a key future development direction for neuroperipheral GNRs systems.

Beyond liposomes, other nanomaterials with high photothermal conversion efficiencies are being explored. A microgel loaded with ropivacaine, combined with graphene oxide, achieved analgesic effects lasting up to 9.5 h upon NIR triggering [84]. Similarly, hybrid nanoparticles leveraging the photothermal properties of copper sulfide and the thermo-responsiveness of a smart polymer enabled controlled release of bupivacaine at predetermined time points in vitro [85]. Moreover, sophisticated systems like 2D silicene-based mesoporous nanomedicine not only enabled NIR-triggered ropivacaine release but also demonstrated the ability to modulate pain pathways by suppressing c-Fos and TRPV1 expression in dorsal root ganglia [86]. These diverse platforms underscore the broad potential of photothermal materials in creating the next generation of intelligent LAs.

#### 4.2.2. Ultrasound-Triggered Drug Release

While NIR light has been extensively studied, its application for triggering LAs in deep tissues remains challenging. The efficacy of NIR may be influenced by patient-specific factors such as edema, obesity, hematoma, and the depth of the target nerve, in addition to tissue penetration limitations inherent to specific wavelengths [87]. Furthermore, tissue damage may occur at high laser irradiance [88], underscoring the need for a truly non-invasive and deep-penetrating delivery method. Diagnostic and therapeutic ultrasound, a primary tool in clinical anesthesiology for procedures like nerve blocks, offers a promising alternative due to its excellent safety profile, deep tissue penetration capability, and non-invasive nature [89]. Ultrasound-sensitive nanoformulations leverage this energy for on-demand drug release, creating a powerful platform for spatially and temporally controlled analgesia.

The mechanisms of ultrasound-triggered release primarily involve acoustic cavitation (the formation and oscillation of microbubbles) and local thermal effects, which can disrupt carrier integrity or activate responsive materials. For example, the acoustic sensitizer protoporphyrin IX generates reactive oxygen species upon ultrasound stimulation. This burst of ROS subsequently triggers the release of tetrodotoxin from liposomes, initially providing 34.5 ± 5.0 h of analgesia, along with three additional on-demand releases providing an extra 3.2 h of nerve block [90].

Beyond small molecules, complex nanoparticle structures have been engineered to exhibit ultrasonic responsiveness. Hollow mesoporous organosilica nanoparticles (HMONs) degrade upon ultrasound irradiation due to the cleavage of 1O_2_-responsive bridging structures within their framework [91]. This degradation mechanism has been successfully applied to release LAs, providing approximately 6 h of analgesia [92]. Similarly, dendritic mesoporous silica nanoparticles loaded with the ultrasound-sensitive phase-change material perfluoropentane and levobupivacaine provide sustained analgesia for up to 9 h upon stimulation [93].

An innovative approach involves phase-transition nanodroplets. Pentobarbital-loaded decafluorobutane-core nanodroplets can be converted into microbubbles through ultrasound application (58 MHz) within the motor cortex. This phase-change process facilitates both localized drug release and sustained delivery, enabling precise regional brain anesthesia [94]. These diverse systems highlight the multifunctionality of ultrasound as a trigger and its significant potential for clinical translation in on-demand pain management.

#### 4.2.3. Thermally Triggered Drug Release

Thermally responsive systems represent a foundational strategy in the field of smart drug delivery. While thermosensitive materials are frequently combined with photothermal or ultrasonic technologies to achieve external control, formulations that respond solely to intrinsic temperature changes at the site of administration are themselves successful and clinically valuable nanotechnology applications suitable for local anesthesia. These systems typically leverage a sol–gel phase transition at a critical temperature. They are administered as low-viscosity solutions that can be easily injected through a standard syringe [95]. Upon encountering body temperature at the target site (e.g., perineurally), they rapidly undergo a phase transition to form a semi-solid gel depot. A prominent example is a bupivacaine-loaded gel fabricated from the triblock copolymer PLGA-PEG-PLGA, which forms a stable matrix around the sciatic nerve upon injection, providing sustained analgesia [96]. This principle was further demonstrated by a thermoresponsive mixed micellar nanogel composed of lidocaine and prilocaine [97], as well as by the bupivacaine hydrogels that form in vivo, delivering analgesia for up to three days postoperatively [98].

The versatility of this approach stems from the diversity of thermosensitive polymer materials:

- Poly(N-isopropylacrylamide) (PNIPAM) and its copolymers, known for exhibiting a distinct low critical solution temperature near physiological temperatures.
- Poly(ethylene oxide)/poly(propylene oxide) (PEO/PPO) block copolymers (e.g., Pluronics^®^).
- Poly(ε-caprolactone)-poly(ethylene glycol) (PCL-PEG)-based copolymers [99].
- Various other synthetic and natural polymers [100].

These materials can be tailored to exhibit specific gelation temperatures, drug release rates, and degradation characteristics, making them suitable for a wide range of therapeutic applications. The successful application of these thermoresponsive sustained-release nanomedicines in LAs underscores their promising potential for achieving prolonged analgesia with a single-injection, without requiring complex external triggering devices.

#### 4.2.4. Magnetically Triggered Drug Release

Magnetic nanoparticles (MNPs), particularly those based on iron oxides, have garnered significant attention in drug delivery due to their established safety profile and unique responsiveness to external magnetic fields [101]. While their application in targeted regional chemotherapy for cancer has been well-established [102], the principles of magnetic guidance and activation are equally suitable for local anesthesia. The ability to spatially control and retain drug carriers at a desired site using a magnet offers a compelling strategy to enhance the specificity and duration of nerve block.

The therapeutic application of MNPs in anesthesia is achieved through magnetic targeting and magnetic hyperthermia. In the first approach, MNPs conjugated with anesthetics can be physically concentrated and retained around target nerves under a static magnetic field, effectively forming a localized drug reservoir. Early studies demonstrated the concept, demonstrating that magnetite nanoparticles conjugated with anesthetics produced positive analgesic effects [103]. Safety evaluation revealed that lidocaine-loaded magnetic nanoparticles (FeAu@gelatin-lidocaine complexes) did not release lidocaine in the absence of high-frequency induction wave stimulation, nor did they exhibit significant cytotoxicity. These findings demonstrate the favorable safety profile of this nanoplatform [104]. Furthermore, a bupivacaine-loaded magnetic nanogel polymer was shown to exhibit superior sustained-release properties, particularly under specific physiological conditions such as low temperature and pH [105].

The second mechanism leverages the hyperthermic effect of superpara MNPs. When exposed to an alternating magnetic field (AMF), these nanoparticles generate localized heat. This thermal energy can then be used to trigger drug release from a thermosensitive carrier. For instance, lidocaine encapsulated within iron–gold alloy nanoparticles (FeAu NPs) was successfully released upon exposure to high-frequency induction waves, demonstrating a clean, externally controlled on-demand release system [106]. This combination of magnetic targeting for spatial control and magnetic hyperthermia for temporal control positions MNPs as a versatile and powerful platform for the next generation of intelligent LAs.

#### 4.2.5. Emerging and Multi-Responsive Nano-Preparations

Beyond known triggering methods such as light, ultrasound, heat, and magnetism, several alternative and emerging drug release approaches are being explored. Examples include electrochemical triggering and the use of low-power blue light-emitting diodes (LEDs) [107], which offer advantages in achieving precise spatial and temporal control with minimal tissue interaction.

A highly promising research direction lies in designing multi-responsive nano-formulations that integrate two or more triggering mechanisms (e.g., light and magnetic, ultrasound and thermal, or chemical and electrical). By combining different physical stimuli or incorporating chemical responsiveness (e.g., to pH or enzymes), these sophisticated systems offer enhanced controllability, greater redundancy, and the ability to adapt to intricate physiological environments. This makes them highly valuable for achieving sophisticated on-demand analgesic profiles.

It is important to note that research into these novel delivery methods for LAs remains in preliminary stages [24]. Current explorations face several challenges, including limited depth in mechanism studies and the need for comprehensive in vivo safety and efficacy data. Nevertheless, the exploration of these innovative formulations is entirely justified, as they hold the key to achieving unprecedented precision in pain management and represent the cutting edge of intelligent drug delivery technology.

### 4.3. Precise Control of Release Kinetics: From “On-Off” to “Dimmer Switch”

While demonstrating the stimulus-responsive “on-off” release mechanism is a critical first step, the ultimate goal of intelligent drug delivery is to achieve precise, spatiotemporal control over release kinetics. This chapter delves into the parameters that enable fine-tuning control, thereby facilitating personalized analgesic regimens that align with the dynamic characteristics of pain.

#### 4.3.1. Release Kinetic Models: Zero-Order, First-Order, and Pulsatile Release

The release profiles of LAs from carriers are not arbitrary but follow definable kinetic models, each with distinct clinical implications. Zero-order kinetics provide a constant release rate, making them ideal for maintaining stable plasma concentrations and a steady-state nerve block, which is highly desirable for prolonged surgical procedures [108]. This is typically achieved by matrix systems where drug diffusion serves as the rate-limiting step. In contrast, first-order kinetics exhibit an exponential decay in release rate, commonly seen in reservoir-type systems like liposomes, resulting in progressively diminishing analgesic effect over time. For on-demand analgesia, pulsatile or burst release is the target, characterized by sharp, high-amplitude drug release events in direct response to a trigger. The ability to design carriers to follow a specific model is fundamental to achieving controlled release [109].

#### 4.3.2. Tuning Release via Material Parameters

The rate of drug release is profoundly influenced by the physicochemical properties of the nanocarrier. In hydrogel-based systems, the cross-linking density directly determines the mesh size of the polymer network. Higher cross-linking density forms a smaller mesh size, which slows drug diffusion, promotes zero-order release and reduces passive leakage. Conversely, a looser network facilitates faster, burst-like release upon triggering [110].

Drug-carrier interactions represent another factor influencing drug release. The strength of non-covalent interactions (e.g., hydrophobic, electrostatic) between the drug molecules and the carrier matrix acts as a secondary retention effect. Stronger interactions can delay initial release and require greater stimulation to trigger, thereby enabling the programming of release thresholds [111].

#### 4.3.3. Tuning Release via Triggering Parameters

Beyond material design, the release profile can be dynamically modulated in real-time by adjusting the stimulus parameters, offering a powerful tool for personalized dose setting.

Energy Intensity/Dose: The magnitude of release events can typically be controlled by the intensity of the stimulus. For example, higher laser power or ultrasound intensity generally generates more heat or cavitation, leading to increased drug release per triggering event [20].

Duration and Frequency: The duration of stimulus application directly correlates with the total drug amount released per event. Furthermore, controlling the frequency of trigger applications enables the delivery of multiple discrete analgesic doses from a single injection, effectively managing intermittent or breakthrough pain.

#### 4.3.4. The Effect of Protein Corona on Triggered Drug Release

The regulation of drug release from nanoformulations depends not only on their intrinsic properties but also on the influence of the target environment. Upon injection into the body and contact with bodily fluids, the surface of nanocarriers rapidly adsorbs proteins from interstitial fluid and plasma, forming a dynamic layer known as the “protein corona”. This process occurs within seconds to minutes and is virtually unavoidable [112]. First, high-abundance proteins (albumin, immunoglobulins, fibrinogen) adsorb to form a “soft corona”. Over time, low-abundance, high-affinity proteins gradually displace them (Vroman effect), forming a “hard corona” [113]. Nanocarriers for regional anesthesia are typically injected around nerves, where these areas are rich in extracellular matrix proteins (collagen, laminin, fibronectin) and inflammation-related proteins (if tissue damage is present). The protein crown formed by these proteins alters the physicochemical properties of the nanocarriers, including particle size, surface charge, surface chemistry, and optical properties. More importantly, the protein coat can form an additional diffusion barrier, delaying drug release [114].

The interference of protein coronas with various triggering mechanisms cannot be overlooked. For external physical triggers, such as photothermal materials represented by gold nanorods, the formation of protein coronas alters the surface properties of nanoparticles, thereby affecting their photothermal conversion efficiency. Bionic modifications mimicking red blood cell membranes have been proven to effectively neutralize surface charges on nanoparticles and improve colloidal stability, indirectly confirming the significant impact of protein coronas on surface properties [115]. For internal chemical triggers, the buffering capacity of the protein coat may attenuate local pH changes, delaying the response of pH-sensitive carriers. Simultaneously, the protein coat may obscure enzyme recognition sites or neutralize trigger enzymes through inhibitors within the coat. Mucin-derived protein coats have been shown to mask, displace, and weaken the active targeting effects of transferrin-modified nanoparticles [116]. More intractably, protein coat formation is a dynamic process exhibiting substantial interindividual variability: patient-specific variables (biological sex, genetic background, disease state, age) directly influence coat composition and behavior; disease states (e.g., diabetes, cancer) alter circulating proteomes, leading to coat composition differences; and the protein composition of the neuroperipheral microenvironment varies by injection site and tissue injury severity [117]. This implies that two patients injected with the same batch of trigger-induced local anesthetic nanoparticle formulations may exhibit entirely different release behaviors due to individual variations in protein crowns. This irreproducibility represents one of the key obstacles to the clinical translation of trigger-induced nanoparticle formulations.

Most trigger-release studies have been validated only in simple buffers, lacking release data in protein-containing media. Protein corona research has predominantly focused on intravenous administration, with minimal attention to the “tissue fluid protein corona” in local delivery. Recent perspectives emphasize that protein corona formation in local delivery (e.g., mucosal tissues) differs significantly from systemic administration, yet relevant studies remain extremely limited [118]. Systematic investigations into trigger efficiency and the structure-function relationship of protein coronas are particularly scarce [119]. Future research should advance in the following directions: establish physiologically relevant release media and validate trigger-release performance at least in systems containing serum or tissue homogenates; develop anti-protein corona strategies such as dense PEG layers or biomimetic membrane coatings; conduct personalized protein corona prediction by integrating patient proteome data to establish predictive models for trigger efficiency; and most critically, perform specialized studies on the neural microenvironment to elucidate its unique protein corona formation characteristics. Existing research indicates that the development of neuropathy is closely linked to protein-coated immunorecognition [120]. Only through deep understanding and active regulation of protein coats can trigger-based local anesthetic nanomedicines truly transition from “in vitro concept” to “in vivo reliability.”

#### 4.3.5. Strategies for Multi-Responsive Systems

To navigate the complex in vivo environment and achieve precise control, integrating multiple triggering logics into a single platform represents a frontier research area. Multi-responsive systems can be designed using two distinct operational modes, including “AND-gate” logic (requiring simultaneous stimuli) and “sequential” logic (requiring programmed order of stimuli).

Systems can be designed where release requires the simultaneous presence of two stimuli. For instance, a nanocarrier might only release its payload in the presence of both mild heat (from a photothermal effect) and a slightly acidic pH (found in inflamed tissues). This AND-gate logic dramatically enhances targeting specificity and safety [121]. In tumor research, it functions as an AND logic gate, requiring both low pH and esterase to be present simultaneously for the release of antitumor drugs. This dual-condition activation ensures that payload release is strictly confined to the specific areas, minimizing systemic exposure.

More advanced systems utilize one stimulus to initiate or activate the carrier, while another stimulus triggers the actual release process. This allows for complex, programmable delivery regimens unattainable with a single trigger, paving the way for truly adaptive analgesic therapies [122]. For instance, drugs can be locally released or confined in a gel state through thermal triggering, with subsequent remote modulation of release rates enabled by externally triggered ultrasound modulation. Based on this principle, multi-response systems hold particular promise. It is even possible to design nanoformulations with triple responsiveness to pH, NIR, and temperature, coordinating multiple stimuli to achieve precise spatial and temporal control.

For the delivery of LAs, multi-response systems offer unique advantages. For instance, a “AND-gate” design can require simultaneous fulfillment of near-infrared light exposure and pH conditions to trigger analgesic effects, ensuring drug release only when and where needed. Additionally, sequential systems may first use an initial magnetic field to guide nanoparticles to target nerves, followed by ultrasound-triggered release for on-demand pain control. These concepts can be directly applied to designing smarter, safer perineural delivery systems for postoperative pain management. To facilitate reader understanding and material selection, Table 2 provides a comprehensive comparison of several mainstream LAs RTDDS, detailing their unique physicochemical mechanisms of action along with their corresponding advantages and disadvantages.

## 5. Bridging the Gap: From Bench to Bedside in Intelligent Local Anesthesia

**Figure 3:**
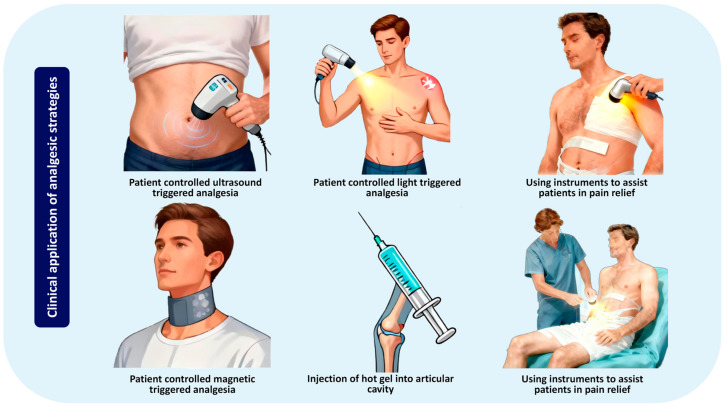
Applications of externally triggered local anesthetic nanoformulations. Ultrasound triggering. Stimulus: focused ultrasound waves from a transducer. Clinical Scenario: Ultrasound-guided nerve block for deep-seated nerves (e.g., transversus abdominis plane block). NIR light triggering. Stimulus: A beam of NIR light. Clinical Scenario: Patient-controlled, non-invasive analgesia for superficial nerves (e.g., post-operative shoulder pain). Clinical scenario: Non-invasive superficial nerve analgesia (such as postoperative shoulder pain) implemented by others to assist patients. Magnetic triggering. Stimulus: Static magnetic field for targeting; alternating magnetic field (AMF) for hyperthermia-triggered release. Clinical scenario: Spatial targeting and on-demand release for complex anatomical regions (e.g., brachial plexus, neck thyroid surgery). Thermal triggering. Stimulus: Ambient body heat or an external mild heat source. Clinical scenario: Injectable depot for prolonged analgesia at routine surgical sites (e.g., Hip and knee joint). Assisting patients with abdominal pain relief by others. The authors created this figure using WPS Presentation (12.1.0.24657).

As shown in Figure 3, intelligent anesthesia systems have broad application prospects in clinical practice. Although the remotely triggered nano-systems for local anesthesia have demonstrated convincing proof-of-concept in preclinical studies, their clinical translation remains a formidable challenge. The journey from promising laboratory findings to a commercially available therapeutic faces numerous scientific, regulatory, and practical hurdles that must be systematically addressed.

### 5.1. Clinical Translation Challenges

There is a significant gap between the idealized performance in research settings and the requirements for clinical use [123]. Many current systems lack the precision control, long-term stability, and reliable repeatability necessary for safe human application. The “on-demand” release profile can be interfered with by patient-specific factors such as age, gender, and comorbidities (e.g., diabetes, hypertension), which may alter local tissue physiology and drug pharmacokinetics. Furthermore, the inter-and intra-patient variability in nerve block site, depth, and drug uptake rates presents a major challenge for standardizing dosage, necessitating a goal-oriented adjustment of nanoparticle design for different anatomical targets.

#### 5.1.1. Biocompatibility and Long-Term Safety

The long-term toxicology, immunogenicity, and clearance pathways of nanomaterials and their degradation products represent the primary safety concerns. While microbial contamination is a managed risk in Good Manufacturing Practice (GMP), the intrinsic immune reactivity of nano-agent components is a more insidious issue that can lead to organism sensitization or chronic inflammation. The use of materials with benign, native body components or those with well-defined hepatic/renal clearance pathways can minimize these risks, though special caution is required for patients with pre-existing organ impairment.

#### 5.1.2. Manufacturing, Quality Control, and Regulatory Pathways

The complexity of these “smart” formulations poses significant challenges in scalable manufacturing, batch-to-batch consistency, and sterility assurance. Moreover, as combination products (drug + device), they face a complex regulatory landscape. Agencies like the FDA require rigorous demonstration of both the safety/efficacy of the drug component and the reliability of the triggering device, creating a steeper path to approval compared to conventional drugs.

#### 5.1.3. Dosage Control and Patient Usability

Quantifying real-time drug release around human nerves remains a technical hurdle, as current validation is largely limited to in vitro or animal models. This uncertainty, combined with the risk of explosive drug release from improper handling, poses a significant safety threat. A two-pronged approach is needed: developing advanced human-compatible monitoring techniques and designing failsafe external triggering devices. These devices should incorporate safety protocols, such as dose-locking mechanisms and maximum dose limits, inspired by the safety features of modern patient-controlled analgesia (PCA) pumps.

### 5.2. A Blueprint for Next-Generation Intelligent Analgesia

Despite these challenges, the future of intelligent LAs is bright, provided research pivots towards more sophisticated, clinically aware designs.

- Closed-Loop Feedback Systems: Future systems should evolve from “open-loop” to “closed-loop” by integrating biosensors that monitor biomarkers of pain (e.g., local pH, inflammatory cytokines). This will enable autonomous, on-demand drug release, truly personalizing the analgesic experience.
- Multifunctional Synergistic Platforms: Moving beyond mere analgesia, next-generation platforms could combine analgesic, anti-inflammatory, antimicrobial, and tissue-repair functionalities. This holistic approach would not only manage pain but also actively promote the healing process.
- Personalized Medicine via Advanced Fabrication: Technologies like 3D printing could be used to create customized implants tailored to a patient’s specific anatomy and projected pain trajectory, offering unprecedented control over drug release profiles.
- Exploration of Novel Materials: Addressing protein corona formation or reducing its interference with drug release from nanoformulations is crucial. Additionally, identifying safer, more responsive, and fully biodegradable materials is paramount. New polymers and inorganic composites with improved biocompatibility and sharper stimulus-responsiveness will form the foundation of future clinical systems.

In conclusion, despite the challenges ahead, the superior cost-effectiveness and enhanced patient experience offered by smart LAs in pain management provide a powerful incentive to overcome these obstacles.

## 6. Conclusions

Local anesthesia, with its long-standing history as a cornerstone of clinical analgesia, continues to drive innovative research aimed at optimizing perioperative recovery. Despite its proven benefits, conventional LAs exhibit significant limitations, often leading to insufficient analgesia or undesirable motor block that directly impairs the quality of postoperative recovery.

The advent of intelligent local anesthetic nanoformulations represents a shift in pain management. Preclinical studies have demonstrated their potential for on-demand, prolonged, and satisfactory analgesia, with continuous improvements in trigger sensitivity and reusability. However, the translation of these promising systems from laboratory to clinic faces formidable challenges. The critical hurdles are no longer merely proof of triggerable release but include precise control over drug release kinetics, the long-term stability of the carriers, and the unequivocal demonstration of their safety and efficacy in human physiology. 

To bridge this gap, we recommend a concerted push for early-phase clinical studies that prioritize patient safety, aimed at generating robust human data to define clinical efficacy. The complexity of pain states may be best addressed by designing multi-responsive platforms that synergistically combine different nanomaterials to enhance stability and responsiveness.

Looking ahead, the clinical translation of intelligent LAs hinges on addressing three pivotal aspects: the consistency of each released dose, the reliability over multiple trigger cycles, and the user-friendliness of the triggering devices. Our ultimate goal remains the development of an ideal smart local anesthetic that delivers truly patient-specific, on-demand analgesia with minimal intervention, thereby fully realizing the promise of ERAS protocols.

## Figures and Tables

**Table 1:** Comparative analysis of current local anesthesia modalities and the evolution toward intelligent nanoformulations.

Anesthesia Modality & Nanoformulations	Mechanism of Action & Target	Major Limitations & Challenges	Therapeutic Objectives of Nanoformulations
Topical Anesthesia	Acts on mucosal/skin surfaces, blocking terminal nerve endings [32].	Limited depth of action; drug dilution/removal by body fluids [35].	Enhance mucoadhesion and tissue penetration for improved surface efficacy [35,36].
Infiltration Anesthesia	Injected into subcutaneous/target tissues, blocking regional nerve endings [37].	Rapid drug dispersion, high doses required; risk of systemic toxicity [38].	Passive targeting: Employ liposomes, microspheres for sustained release, reducing dose frequency and toxicity [39,40,41].
Nerve Block	Injected peripherally into nerve trunks, blocking impulse conduction [45].	Risk of procedural failure, nerve injury, or toxic reactions [46,47,48].	Passive Targeting + Spatial Control: Combining ultrasound technology for precise positioning, it achieves the combination of sustained release with enhanced perineural retention [48,49,50,51].
Intraspinal Anesthesia	Injected into the subarachnoid or epidural space, acting on spinal nerve roots [52].	Hypotension, respiratory depression; catheter-related infection/technical complexity.	Prolong analgesia via sustained-release formulations administered epidurally. Reduction in complications in obstetric anesthesia [52,53].
Conventional Sustained-Release Nanoformulations (e.g., Liposomal Bupivacaine)	Passive diffusion following a predetermined release profile.	“One-time” sustained release; Inflexible; cannot adjust to individual variability or dynamic pain states; ineffective for on-demand analgesia.	Overcoming the “One-time” Limitation. Providing responsive, on-demand pain control.
Intelligent Nanoformulations (Future Direction)	On-demand; release therapeutic agents in response to specific external or internal stimuli [54,55]. active and passive triggering systems [58].	The active triggering system needs to have unique and exploitable physiological signals for pathological conditions. The clinical application of the passive triggering system faces challenges.	Non-invasive or minimally invasive, featuring a trigger-release mechanism with high sensitivity and excellent repeatability and enabling precise control over the dose and duration of each release event

**Table 2:** Comprehensive comparison of triggerable local anesthetic systems.

Trigger Modality	Representative Materials	Mechanism	Advantages	Limitations	Efficacy in Animal Models	Clinical Translation Potential
Light (NIR)	Gold nanorods, Polypyrrole, Pd-phthalocyanines	Photothermal effect: light absorption generates heat, inducing phase transition or structural disruption in the carrier.	High spatiotemporal precision; remote, non-invasive control; tunable tissue penetration.	Limited tissue penetration depth; potential thermal damage at high power; requires line-of-sight access.	Prolonged nerve block (e.g., >37 h with TTX) with multiple on-demand cycles (e.g., 3–4 triggers) [74,76].	Moderate. Promising for superficial nerves but limited by depth penetration. Patient-friendly LED devices could facilitate translation.
Ultrasound	Microbubbles, Hollow mesoporous silica, Perfluorocarbon nanodroplets	Cavitation effect: microbubble oscillation/microjet disrupts carriers. Thermal effect: localized mild heating.	Deep tissue penetration; high safety profile; widely available clinical equipment.	Relatively lower spatiotemporal precision than light; potential for non-specific bio-effects (e.g., cavitation).	Significant extension of analgesia (e.g., >34 h baseline + 3.2 h triggered) with multiple releases [90,93].	High. Excellent alignment with existing ultrasound-guided regional anesthesia practice; considered one of the most translatable modalities.
Thermal	PNIPAM, PLGA-PEG-PLGA, PCL-PEG copolymers	Thermal phase transition: polymer matrix undergoes sol–gel transition or change in permeability upon heating.	Simple principle; can be triggered by body heat alone (forming a depot) or an external source; sustained release profile.	Limited on/off cycling capability; slow response kinetics; difficult to achieve deep, localized heating without an energy mediator.	Formation of a stable depot providing sustained analgesia for days (e.g., 3 days post-operation) [96,98].	High (for body heat). Injectable thermogels are in clinical trials for other drugs, paving the way for anesthetics. External thermal control has lower potential.
Magnetic	Iron oxide nanoparticles (Fe_3_O_4_), Iron–gold alloys (FeAu)	1. Targeting: Spatial retention under a static magnetic field. 2. Triggering: Heat generation under an alternating magnetic field (AMF) induces release.	Deep penetration of magnetic fields; dual functionality (targeting + triggering); excellent safety profile of iron oxides.	Requires specialized magnetic equipment; potential concerns with long-term tissue retention of metal-based particles.	Prolonged duration (e.g., 14-fold increase in safety index) and targeted delivery demonstrated [104,106].	Moderate to Low. Targeting is promising, but the clinical infrastructure for applying high-frequency AMF is not yet widespread.

Abbreviation: NIR, Near-Infrared; TTX, Tetrodotoxin; PNIPAM, Poly(N-isopropylacrylamide); PLGA-PEG-PLGA, Poly(lactic-co-glycolic acid)-block-poly(ethylene glycol)-block-poly(lactic-co-glycolic acid); PCL-PEG, Poly(ε-caprolactone)-poly(ethylene glycol); AMF, Alternating Magnetic Field.

## Data Availability

No new data were created or analyzed in this study.

## Abbreviations

The following abbreviations are used in this manuscript:

LAs	Local anesthetics
ERAS	Enhancing recovery after surgery
NDCs	Nanoscale drug carriers
RTDDS	Remotely triggerable drug delivery systems
NIR	Near-infrared
GNRs	Gold nanorods
MNPs	Magnetic nanoparticles

## References

[1] Scholz A. (2002). Mechanisms of (local) anaesthetics on voltage-gated sodium and other ion channels. Br. J. Anaesth..

[2] Dewachter P., Mouton-Faivre C., Emala C.W. (2009). Anaphylaxis and Anesthesia: Controversies and new insights. Anesthesiology.

[3] Lirk P., Picardi S., Hollmann M.W. (2014). Local anaesthetics: 10 essentials. Eur. J. Anaesthesiol..

[4] Woloszczuk-Gebicka B., Grabowski T., Borucka B., Karas-Trzeciak M. (2014). Pharmacokinetics of sufentanil administered with 0.2% ropivacaine as a continuous epidural infusion for postoperative pain relief in infants. Pediatr. Anesth..

[5] Ilfeld B.M. (2017). Continuous Peripheral Nerve Blocks: An Update of the Published Evidence and Comparison With Novel, Alternative Analgesic Modalities. Anesth. Analg..

[6] Min B.M., Kim J.H. (2013). Epidural catheterization with a subcutaneous injection port for the long-term administration of opioids and local anesthetics to treat zoster-associated pain -a report of two cases-. Korean J. Anesthesiol..

[7] Thomas F., Drolet P., Varin F. (2011). Simultaneous percutaneous implantation of a microdialysis probe for monitoring perineural concentrations of local anaesthetics during peripheral nerve block in rabbits. Vet. Anaesth. Analg..

[8] Zink W., Seif C., Bohl E., Hacke N., Braun P.M., Sinner B., Martin E., Fink A.R.H., Graf B.M. (2003). The Acute Myotoxic Effects of Bupivacaine and Ropivacaine After Continuous Peripheral Nerve Blockades. Anesth. Analg..

[9] Nichols J.W., Sakurai Y., Harashima H., Bae Y.H. (2017). Nano-sized drug carriers: Extravasation, intratumoral distribution, and their modeling. J. Control. Release.

[10] Li Z., Li G., Xu J., Li C., Han S., Zhang C., Wu P., Lin Y., Wang C., Zhang J. (2022). Hydrogel Transformed from Nanoparticles for Prevention of Tissue Injury and Treatment of Inflammatory Diseases. Adv. Mater..

[11] Saeed M., Chen F., Ye J., Shi Y., Lammers T., De Geest B.G., Xu Z.P., Yu H. (2021). From Design to Clinic: Engineered Nanobiomaterials for Immune Normalization Therapy of Cancer. Adv. Mater..

[12] Yu J., Qian C., Zhang Y., Cui Z., Zhu Y., Shen Q., Ligler F.S., Buse J.B., Gu Z. (2017). Hypoxia and H_2_O_2_ Dual-Sensitive Vesicles for Enhanced Glucose-Responsive Insulin Delivery. Nano Lett..

[13] da Silva A., Lepetre-Mouelhi S., Couvreur P. (2022). Micro- and nanocarriers for pain alleviation. Adv. Drug Deliv. Rev..

[14] Weldon C., Ji T., Nguyen M.-T., Rwei A., Wang W., Hao Y., Zhao C., Mehta M., Wang B.Y., Tsui J. (2018). Nanoscale Bupivacaine Formulations To Enhance the Duration and Safety of Intravenous Regional Anesthesia. ACS Nano.

[15] Reis A.V., Guilherme M.R., Mattoso L.H.C., Rubira A.F., Tambourgi E.B., Muniz E.C. (2008). Nanometer- and Submicrometer-Sized Hollow Spheres of Chondroitin Sulfate as a Potential Formulation Strategy for Anti-inflammatory Encapsulation. Pharm. Res..

[16] Rennó C.C., Papini J.Z.B., Cereda C.M.S., Martinez E., Montalli V.A., de Paula E., Júnior J.P., Calafatti S.A., Tofoli G.R. (2019). Preclinical Evaluation of Ropivacaine in 2 Liposomal Modified Systems. Anesth. Analg..

[17] Ilfeld B.M., Eisenach J.C., Gabriel R.A. (2020). Clinical Effectiveness of Liposomal Bupivacaine Administered by Infiltration or Peripheral Nerve Block to Treat Postoperative Pain. Anesthesiology.

[18] Liu Q., Santamaria C.M., Wei T., Zhao C., Ji T., Yang T., Shomorony A., Wang B.Y., Kohane D.S. (2017). Hollow Silica Nanoparticles Penetrate the Peripheral Nerve and Enhance the Nerve Blockade from Tetrodotoxin. Nano Lett..

[19] Alejo T., Sebastian V., Mendoza G., Arruebo M. (2022). Hybrid thermoresponsive nanoparticles containing drug nanocrystals for NIR-triggered remote release. J. Colloid Interface Sci..

[20] Cao Y., Dumani D.S., Hallam K.A., Emelianov S.Y., Ran H. (2023). Real-time monitoring of NIR-triggered drug release from phase-changeable nanodroplets by photoacoustic/ultrasound imaging. Photoacoustics.

[21] Chen W.-H., Luo G.-F., Vázquez-González M., Cazelles R., Sohn Y.S., Nechushtai R., Mandel Y., Willner I. (2018). Glucose-Responsive Metal–Organic-Framework Nanoparticles Act as “Smart” Sense-and-Treat Carriers. ACS Nano.

[22] Xia H., Qin M., Wang Z., Wang Y., Chen B., Wan F., Tang M., Pan X., Yang Y., Liu J. (2022). A pH-/Enzyme-Responsive Nanoparticle Selectively Targets Endosomal Toll-like Receptors to Potentiate Robust Cancer Vaccination. Nano Lett..

[23] He Y., Qin L., Huang Y., Ma C. (2020). Advances of Nano-Structured Extended-Release Local Anesthetics. Nanoscale Res. Lett..

[24] Zhao M., Zhou M., Lu P., Wang Y., Zeng R., Liu L., Zhu S., Kong L., Zhang J. (2024). Local anesthetic delivery systems for the management of postoperative pain. Acta Biomater..

[25] Ma H., Pan Z., Lai B., Zan C., Liu H. (2023). Recent Research Advances in Nano-Based Drug Delivery Systems for Local Anesthetics. Drug Des. Dev. Ther..

[26] Guo B., Zhou Q., Zhang X., Ma Q., Ma M., Wang T. (2026). Recent advances in local anesthetic drug delivery systems based on natural polymers. Front. Bioeng. Biotechnol..

[27] Lee K.K., Jeong W., Chae M. (2025). Hydrogel Drug Delivery Systems and Liposomal Bupivacaine: Innovations and Future Perspectives in Pain Management. J. Clin. Med..

[28] Jeong J.-O., Kim M., Kim S., Lee K.K., Choi H. (2025). Advanced Hydrogel Systems for Local Anesthetic Delivery: Toward Prolonged and Targeted Pain Relief. Gels.

[29] Gardhouse S., Sanchez A. (2022). Rabbit Sedation and Anesthesia. Vet. Clin. N. Am. Exot. Anim. Pract..

[30] Wang J., Hou X., Zhang X., Wang X., Qin W., Li Q., Ma F., Sun L. (2023). Comparison of pulmonary function during interscalene block vs. supraclavicular block: A single-center, double-blind, randomized trial. BMC Anesthesiol..

[31] Debry N., Delhaye C., Azmoun A., Ramadan R., Fradi S., Brenot P., Sudre A., Moussa M.D., Tchetche D., Ghostine S. (2016). Transcarotid Transcatheter Aortic Valve Replacement: General or Local Anesthesia. JACC Cardiovasc. Interv..

[32] Doyle D.J. (2015). Airway anesthesia: Theory and practice. Anesthesiol. Clin..

[33] Shrimpton A.J., O’FArrell G., Howes H.M., Craven R., Duffen A.R., Cook T.M., Reid J.P., Brown J.M., Pickering A.E. (2023). The Bristol Awake Fibre-Optic Intubation Collective. A quantitative evaluation of aerosol generation during awake tracheal intubation. Anaesthesia.

[34] Jee D., Park S.Y. (2003). Lidocaine Sprayed Down the Endotracheal Tube Attenuates the Airway-Circulatory Reflexes by Local Anesthesia During Emergence and Extubation. Anesth. Analg..

[35] Koo C.-H., Kim J., Na H.-S., Ryu J.-H., Shin H.-J. (2022). The effect of lidocaine patch for postoperative pain: A meta-analysis of randomized controlled trials. J. Clin. Anesth..

[36] Williams L.K., Weber J.M., Pieper C.D., Lorenzo A.B., Moss H.M., Havrilesky L.J.M. (2020). Lidocaine–Prilocaine Cream Compared With Injected Lidocaine for Vulvar Biopsy: A Randomized Controlled Trial. Obstet. Gynecol..

[37] Bajwa M.S., Bashir M.M., Bajwa M.H., Iqbal Z., Salahuddin M.A., Hussain A., Shahzad F. (2023). How long to wait after local infiltration anaesthesia: Systematic review. BJS Open.

[38] Neal J.M., Neal E.J., Weinberg G.L. (2020). American Society of Regional Anesthesia and Pain Medicine Local Anesthetic Systemic Toxicity checklist: 2020 version. Reg. Anesth. Pain Med..

[39] Desai N., Kirkham K.R., Albrecht E. (2021). Local anaesthetic adjuncts for peripheral regional anaesthesia: A narrative review. Anaesthesia.

[40] Bay-Nielsen M., Klarskov B., Bech K., Andersen J., Kehlet H. (1999). Levobupivacaine vs bupivacaine as infiltration anaesthesia in inguinal herniorrhaphy. Br. J. Anaesth..

[41] Andersen F.H., Nielsen K., Kehlet H. (2005). Combined ilioinguinal blockade and local infiltration anaesthesia for groin hernia repair—A double-blind randomized study. Br. J. Anaesth..

[42] Callesen T., Bech K., Kehlet H. (1998). The feasibility, safety and cost of infiltration anaesthesia for hernia repair. Hvidovre Hospital Hernia Group. Anaesthesia.

[43] Kwon H., Shin Y., Cho S., Park S., Jung I. (2014). Factors affecting the success rate of buccal infiltration anaesthesia in the mandibular molar region. Int. Endod. J..

[44] Kuivalainen A., Niemi-Murola L., Widenius T., Elonen E., Rosenberg P.H. (2010). Comparison of articaine and lidocaine for infiltration anaesthesia in patients undergoing bone marrow aspiration and biopsy. Eur. J. Pain.

[45] Catalani B., Jones J. (2022). Peripheral Nerve Block Complications in Children. Orthop. Clin. N. Am..

[46] Moga F.X., Galbo M.D.L., Overman D.M., Friedrichsdorf S.J. (2020). Post-Cardiotomy Parasternal Nerve Block with Bupivacaine May Be Associated with Reduced Post-Operative Opioid Use in Children: A Retrospective Cohort Study. Children.

[47] Heydinger G., Tobias J., Veneziano G. (2021). Fundamentals and innovations in regional anaesthesia for infants and children. Anaesthesia.

[48] Dingeman R.S., Barus L.M., Chung H.K., Clendenin D.J., Lee C.S., Tracy S., Johnson V.M., Dennett K.V., Zurakowski D., Chen C. (2013). Ultrasonography-Guided Bilateral Rectus Sheath Block vs Local Anesthetic Infiltration After Pediatric Umbilical Hernia Repair: A prospective randomized clinical trial. JAMA Surg..

[49] Xu C., Gu F., Liu Y., Chen R., Wang C., Lu J. (2022). The median effective analgesic concentration of ropivacaine in ultrasound-guided interscalene brachial plexus block after arthroscopic rotator cuff repair. Front. Pharmacol..

[50] Xue M., Guo C., Han K., Bai R., An R., Shen X. (2022). Analgesia Effect of Ultrasound-Guided Transversus Abdominis Plane Block Combined with Intravenous Analgesia After Cesarean Section: A Double-Blind Controlled Trial. Pain Ther..

[51] Yao Y., Fu S., Dai S., Yun J., Zeng M., Li H., Zheng X. (2020). Impact of ultrasound-guided erector spinae plane block on postoperative quality of recovery in video-assisted thoracic surgery: A prospective, randomized, controlled trial. J. Clin. Anesth..

[52] Krames E.S. (2012). A History of Intraspinal Analgesia, a Small and Personal Journey. Neuromodulation.

[53] Stav M., Matatov Y., Hoffmann D., Heesen P., Gliesche V., Binyamin Y., Ioscovich A., Eidelman L.A., Orbach-Zinger S. (2022). Incidence of conversion to general anaesthesia and need for intravenous supplementation in parturients undergoing caesarean section under spinal anaesthesia: A retrospective observational study. Acta Anaesthesiol. Scand..

[54] Alsehli M. (2020). Polymeric nanocarriers as stimuli-responsive systems for targeted tumor (cancer) therapy: Recent advances in drug delivery. Saudi Pharm. J..

[55] Hou Y., Meng X., Zhang S., Sun F., Liu W. (2022). Near-infrared triggered ropivacaine liposomal gel for adjustable and prolonged local anaesthesia. Int. J. Pharm..

[56] Chu L.-Y., Li Y., Zhu J.-H., Wang H.-D., Liang Y.-J. (2004). Control of pore size and permeability of a glucose-responsive gating membrane for insulin delivery. J. Control. Release.

[57] Zhou Y., Ke P., Bao X., Wu H., Xia Y., Zhang Z., Zhong H., Dai Q., Wu L., Wang T. (2022). Peptide nano-blanket impedes fibroblasts activation and subsequent formation of pre-metastatic niche. Nat. Commun..

[58] Kost J., Langer R. (2001). Responsive polymeric delivery systems. Adv. Drug Deliv. Rev..

[59] Li Y., Owens G.E., Kohane D.S. (2023). Materials for Controlled Release of Local Anesthetics. ChemMedChem.

[60] Bagshaw K.R., Hanenbaum C.L., Carbone E.J., Lo K.W., Laurencin C.T., Walker J., Nair L.S. (2015). Pain Management Via Local Anesthetics and Responsive Hydrogels. Ther. Deliv..

[61] Huang Y., Chen T., Ren C., Bao B., Huang R., Sun Y., Yu C., Yang Y., Wong W.T., Zeng Q. (2025). High-Strength Gelatin Hydrogel Scaffold with Drug Loading Remodels the Inflammatory Microenvironment to Enhance Osteoporotic Bone Repair. Adv. Mater..

[62] Thoma A., Whatmore R., Amstad E. (2025). Microstructured thermo-responsive double network granular hydrogels. Mater. Adv..

[63] Yang J., Zhao Q., Lu B., Lv Y., Jiang W., Chen X., Zhang S., Zhao W., Jiang L., Zhang J. (2025). Injectable thermosensitive hydrogel based on hyaluronic acid and poloxamer for sustained bupivacaine release and prolonged analgesia. Int. J. Biol. Macromol..

[64] Feng Z.-T., Tsai W.-B. (2025). Dual Cross-Linking of Catechol-Alginate Hydrogels: A Strategy for Enhanced Stability and Sustained Drug Delivery. ACS Omega.

[65] Jiang H., Lu X., Bu T., Yang X., Li X., Ren X., Xu X., Fan C., He J., Zhang X. (2025). Mechanics Mediated Semi-Convertible Hydrogel Enabled Sustained Drug Release. Adv. Healthc. Mater..

[66] Savolainen H., Hosseiniyan N., Piedrahita-Bello M., Ikkala O. (2025). Bioinspired nondissipative mechanical energy storage and release in hydrogels via hierarchical sequentially swollen stretched chains. Nat. Commun..

[67] Ma Z., Nelson D.M., Hong Y., Wagner W.R. (2010). Thermally Responsive Injectable Hydrogel Incorporating Methacrylate-Polylactide for Hydrolytic Lability. Biomacromolecules.

[68] Maity S., Mahata K., Meshram B., Banerjee S. (2025). Smart Polymer-Derived Injectable Hydrogels: Current Status and Future Perspectives. ACS Polym. Au.

[69] Salamat Q., Moradi R., Nadizadeh Z., Kavehpour P., Soylak M., Asimov A., Rahman Z., Kovářík T., Babuška V., Deshmukh K. (2026). Chitosan based smart injectable hydrogels for biomedical applications: A comprehensive review. Bioact. Mater..

[70] Nascimento A.T.D., Stoddart P.R., Goris T., Kael M., Manasseh R., Alt K., Tashkandi J., Kim B.C., Moulton E.S. (2025). Stimuli-Responsive Materials for Biomedical Applications. Adv. Mater..

[71] Zhong J., Li Y., Li X., Liang F., Tao R., Qian S., Fan X. (2025). Near Infrared Light-Triggered Small Molecule Chemical Reactions in Biocompatible Systems. Bioconj. Chem..

[72] Rwei A.Y., Lee J.-J., Zhan C., Liu Q., Ok M.T., Shankarappa S.A., Langer R., Kohane D.S. (2015). Repeatable and adjustable on-demand sciatic nerve block with phototriggerable liposomes. Proc. Natl. Acad. Sci. USA.

[73] Fu P., Teng I., Liu W., Chen I., Ho C., Hsing C., Sun C., Hung K. (2022). Association of scalp block with intraoperative hemodynamic profiles and postoperative pain outcomes at 24–48 hours following craniotomy: An updated systematic review and meta-analysis of randomized controlled studies. Pain Pract..

[74] Zhan C., Wang W., Santamaria C., Wang B., Rwei A., Timko B.P., Kohane D.S. (2017). Ultrasensitive Phototriggered Local Anesthesia. Nano Lett..

[75] Dong G., Qiu R., Xu C. (2025). Dexmedetomidine as a Ropivacaine Adjuvant in a Thoracic Paravertebral Block Combined With an Erector Spinae Plane Block for Improving Early Quality of Recovery After Transapical Transcatheter Aortic Valve Implantation. Kaohsiung J. Med. Sci..

[76] Zhan C., Wang W., McAlvin J.B., Guo S., Timko B.P., Santamaria C., Kohane D.S. (2015). Phototriggered Local Anesthesia. Nano Lett..

[77] Rwei A.Y., Zhan C., Wang B., Kohane D.S. (2017). Multiply repeatable and adjustable on-demand phototriggered local anesthesia. J. Control. Release.

[78] Rwei A.Y., Wang B.Y., Ji T., Zhan C., Kohane D.S. (2017). Enhanced Triggering of Local Anesthetic Particles by Photosensitization and Photothermal Effect Using a Common Wavelength. Nano Lett..

[79] Han S., Al-Jamal K.T. (2023). Combined Facile Synthesis, Purification, and Surface Functionalization Approach Yields Monodispersed Gold Nanorods for Drug Delivery Applications. Part. Part. Syst. Charact..

[80] Zhoua Y., Quana G., Wub Q., Zhangc X., Niua B., Wua B., Huanga Y., Pana X., Wuad C. (2018). Mesoporous silica nanoparticles for drug and gene delivery. Acta Pharm. Sin. B.

[81] Mehrizi T.Z., Rezayat S.M., Shahmabadi H.E. (2025). Latest Findings on the Effects of Gold Nanoparticles on the Storage Quality of Blood Products (2011-2022)—A Narrative Review. Curr. Drug Deliv..

[82] Xie L., Zhang X., Chu C., Dong Y., Zhang T., Li X., Liu G., Cai W., Han S. (2021). Preparation, toxicity reduction and radiation therapy application of gold nanorods. J. Nanobiotechnol..

[83] Shi X., Perry H.L., Wilton-Ely J.D.E.T. (2021). Strategies for the functionalisation of gold nanorods to reduce toxicity and aid clinical translation. Nanotheranostics.

[84] Pang Q., Zhao J., Zhang S., Zhang X. (2020). Near-infrared triggered on-demand local anesthesia using a jammed microgels system. J. Biomater. Sci. Polym. Ed..

[85] de Solorzano I.O., Alejo T., Abad M., Bueno-Alejo C., Mendoza G., Andreu V., Irusta S., Sebastian V., Arruebo M. (2019). Cleavable and thermo-responsive hybrid nanoparticles for on-demand drug delivery. J. Colloid Interface Sci..

[86] Yin S., Gao P., Yu L., Zhu L., Yu W., Chen Y., Yang L. (2022). Engineering 2D Silicene-Based Mesoporous Nanomedicine for In Vivo Near-Infrared-Triggered Analgesia. Adv. Sci..

[87] Stolik S., Delgado J., Pérez A., Anasagasti L. (2000). Measurement of the penetration depths of red and near infrared light in human “ex vivo” tissues. J. Photochem. Photobiol. B Biol..

[88] Croke L. (2020). Guideline for laser safety. AORN J..

[89] Albrecht E., Chin K.J. (2020). Advances in regional anaesthesia and acute pain management: A narrative review. Anaesthesia.

[90] Rwei A.Y., Paris J.L., Wang B., Wang W., Axon C.D., Vallet-Regí M., Langer R., Kohane D.S. (2017). Ultrasound-triggered local anaesthesia. Nat. Biomed. Eng..

[91] Fan J., Zhang Z., Wang Y., Lin S., Yang S. (2020). Photo-responsive degradable hollow mesoporous organosilica nanoplatforms for drug delivery. J. Nanobiotechnol..

[92] Gao X., Zhu P., Yu L., Yang L., Chen Y. (2019). Ultrasound/Acidity-Triggered and Nanoparticle-Enabled Analgesia. Adv. Healthc. Mater..

[93] Song X., Luan M., Zhang W., Zhang R., Xue L., Luan Y. (2022). Moderate-Intensity Ultrasound-Triggered On-Demand Analgesia Nanoplatforms for Postoperative Pain Management. Int. J. Nanomed..

[94] Lea-Banks H., O’REilly M.A., Hamani C., Hynynen K. (2020). Localized anesthesia of a specific brain region using ultrasound-responsive barbiturate nanodroplets. Theranostics.

[95] Alexander A., Ajazuddin, Khan J., Saraf S., Saraf S. (2013). Poly(ethylene glycol)–poly(lactic-co-glycolic acid) based thermosensitive injectable hydrogels for biomedical applications. J. Control. Release.

[96] Zhang W., Ning C., Xu W., Hu H., Li M., Zhao G., Ding J., Chen X. (2018). Precision-guided long-acting analgesia by hydrogel-immobilized bupivacaine-loaded microsphere. Theranostics.

[97] Sharma G., Kamboj S., Thakur K., Negi P., Raza K., Katare O.P. (2016). Delivery of Thermoresponsive-Tailored Mixed Micellar Nanogel of Lidocaine and Prilocaine with Improved Dermatokinetic Profile and Therapeutic Efficacy in Topical Anaesthesia. AAPS PharmSciTech.

[98] Mei L., Xie Y., Huang Y., Wang B., Chen J., Quan G., Pan X., Liu H., Wang L., Liu X. (2018). Injectable in situ forming gel based on lyotropic liquid crystal for persistent postoperative analgesia. Acta Biomater..

[99] Dethe M.R., Prabakaran A., Ahmed H., Agrawal M., Roy U., Alexander A. (2022). PCL-PEG copolymer based injectable thermosensitive hydrogels. J. Control. Release.

[100] Klouda L. (2015). Thermoresponsive hydrogels in biomedical applications: A seven-year update. Eur. J. Pharm. Biopharm..

[101] Yu Q., Xiong X.-Q., Zhao L., Xu T.-T., Bi H., Fu R., Wang Q.-H. (2018). Biodistribution and Toxicity Assessment of Superparamagnetic Iron Oxide Nanoparticles In Vitro and In Vivo. Curr. Med. Sci..

[102] Khan S.A., Sharma R. (2023). Super Para-Magnetic Iron Oxide Nanoparticles (SPIONs) in the Treatment of Cancer: Challenges, Approaches, and its Pivotal Role in Pancreatic, Colon, and Prostate Cancer. Curr. Drug Deliv..

[103] Mantha V.R.R., Nair H.K., Venkataramanan R., Gao Y.Y., Matyjaszewski K., Dong H., Li W., Landsittel D., Cohen E., Lariviere W.R. (2014). Nanoanesthesia: A novel, intravenous approach to ankle block in the rat by magnet-directed concentration of ropivacaine-associated nanoparticles. Anesth. Analg..

[104] Mamun A., Sabantina L. (2023). Electrospun Magnetic Nanofiber Mats for Magnetic Hyperthermia in Cancer Treatment Applications-Technology, Mechanism, and Materials. Polymers.

[105] Nadri S., Mahmoudvand H., Eatemadi A. (2016). Magnetic nanogel polymer of bupivacaine for ankle block in rats. J. Microencapsul..

[106] Ting C.-K., Dhawan U., Tseng C.-L., Gong C.-S.A., Liu W.-C., Tsai H.-D., Chung R.-J. (2020). Hyperthermia-Induced Controlled Local Anesthesia Administration Using Gelatin-Coated Iron–Gold Alloy Nanoparticles. Pharmaceutics.

[107] Zhang W., Ji T., Li Y., Zheng Y., Mehta M., Zhao C., Liu A., Kohane D.S. (2020). Light-triggered release of conventional local anesthetics from a macromolecular prodrug for on-demand local anesthesia. Nat. Commun..

[108] Goldmünz E.Y., Aserin A., Pal A., Shimon D., Ottaviani M., Garti N. (2025). pH-sensitive lyotropic liquid crystal beads designed for oral zero-order extended drug release. Int. J. Pharm..

[109] Duan X., Chen H.-L., Guo C. (2022). Polymeric Nanofibers for Drug Delivery Applications: A Recent Review. J. Mater. Sci. Mater. Med..

[110] Feng A., Shi Y., Onggowarsito C., Zhang X.S., Mao S., Johir M.A., Fu Q., Nghiem L.D. (2024). Structure-Property Relationships of Hydrogel-based Atmospheric Water Harvesting Systems. ChemSusChem.

[111] Hoda M., Sufi S.A., Cavuturu B., Rajagopalan R. (2017). Stabilizers Influence Drug–Polymer Interactions and Physicochemical Properties of Disulfiram-Loaded Poly-Lactide-Co-Glycolide Nanoparticles. Future Sci. OA.

[112] Corbo C., Molinaro R., Parodi A., Furman N.E.T., Salvatore F., Tasciotti E. (2015). The Impact of Nanoparticle Protein Corona on Cytotoxicity, Immunotoxicity and Target Drug Delivery. Nanomedicine.

[113] Lins A., Fortmann M., Mulac-Hahnen D., Humpf H.-U., Langer K. (2025). The nanoparticle protein corona impacts interactions with monocytes and macrophages. Int. J. Pharm..

[114] Ma Z., Bai J., Jiang X. (2015). Monitoring of the Enzymatic Degradation of Protein Corona and Evaluating the Accompanying Cytotoxicity of Nanoparticles. ACS Appl. Mater. Interfaces.

[115] Rodrigues C.F., Correia I.J., Moreira A.F. (2024). Red blood cell membrane-camouflaged gold-core silica shell nanorods for cancer drug delivery and photothermal therapy. Int. J. Pharm..

[116] Yang D., Feng Y., Yuan Y., Zhang L., Zhou Y., Midgley A.C., Wang Y., Liu N., Li G., Yao X. (2024). Protein Coronas Derived from Mucus Act as Both Spear and Shield to Regulate Transferrin Functionalized Nanoparticle Transcellular Transport in Enterocytes. ACS Nano.

[117] Cisneros E.P., Morse B.A., Savk A., Malik K., Peppas N.A., Lanier O.L. (2024). The role of patient-specific variables in protein corona formation and therapeutic efficacy in nanomedicine. J. Nanobiotechnol..

[118] Cinar O., Gebologlu I.K., Oncel S.S. (2025). Nanoparticle-Based Therapeutic Strategies in Respiratory Diseases: Current Approaches and Future Perspectives. Thorac. Res. Pract..

[119] Yunusova N.V., Popova N.O., Udintseva I.N., Klyushina T.S., Kazantseva D.V., Smirnova L.P. (2023). The Role of Intravesicular Proteins and the Protein Corona of Extracellular Vesicles in the Development of Drug-Induced Polyneuropathy. Curr. Issues Mol. Biol..

[120] Dietz L., Oberländer J., Mateos-Maroto A., Schunke J., Fichter M., Krämer-Albers E., Landfester K., Mailänder V. (2023). Uptake of extracellular vesicles into immune cells is enhanced by the protein corona. J. Extracell. Vesicles.

[121] Guo F., Li G., Ma S., Zhou H., Chen X. (2019). Multi-Responsive Nanocarriers Based on β-CD-PNIPAM Star Polymer Coated MSN-SS-Fc Composite Particles. Polymers.

[122] He Y., Zhou J., Ma S., Nie Y., Yue D., Jiang Q., Wali A.R.M., Tang J.Z., Gu Z. (2016). Multi-Responsive “Turn-On” Nanocarriers for Efficient Site-Specific Gene Delivery In Vitro and In Vivo. Adv. Healthc. Mater..

[123] Garg A., Agrawal R., Chauhan C.S., Deshmukh R. (2024). In-situ gel: A smart carrier for drug delivery. Int. J. Pharm..

